# Editorial: HIV/AIDS: pathogenesis and vaccine

**DOI:** 10.3389/fcimb.2025.1584301

**Published:** 2025-03-17

**Authors:** Haibo Wang, Xiaofan Lu

**Affiliations:** ^1^ State Key Laboratory of Emerging Infectious Disease Detection, Zhuhai International Travel Healthcare Center, Zhuhai, China; ^2^ Beijing Key Laboratory for HIV/AIDS Research, Sino-French Joint Laboratory for Research on Humoral Immune Response to HIV Infection, Clinical and Research Center for Infectious Diseases, Beijing Youan Hospital, Capital Medical University, Beijing, China

**Keywords:** HIV/AIDS, pathogenesis, immune function, antiretroviral therapy, prevention

Human Immunodeficiency Virus (HIV) severely damages the immune system, with T-lymphocytes being the primary targets. Although antiretroviral therapy (ART) is effective in treating HIV infection, the virus can remain in reservoirs and cause persistent infection, leading to cumulative deleterious effects on immune function. Therefore, it is crucial to characterize both virological and immunological mechanisms associated with the pathogenesis of HIV/AIDS, which would enable us to identify key factors in viral-host interaction, facilitating the development of novel vaccines and other therapeutic strategies.

This Research Topic aims to invite investigators from around the world to contribute original research articles and review articles that will contribute to the ongoing efforts for the elimination of HIV/AIDS. There are a total of six articles in this Research Topic, which include both basic research and clinical studies.

Wang et al. from Bethune International Peace Hospital and Tianjin Second People’s Hospital investigated the function of polymorphonuclear myeloid-derived suppressor cells (PMN-MDSCs) during HIV infection. Although there have been significant advances in the specific roles of MDSCs in HIV/AIDS-related pathological conditions (Ademe, 2020; Yaseen et al., 2021; Ostrand-Rosenberg et al., 2023), their potential implications in the incomplete immune recovery process remain elusive. By exploring the frequency, phenotype, and function of circulating MDSCs in different groups of HIV-1 infected individuals, Wang et al. found that PMN-MDSCs are more abundant in HIV-infected individuals and can suppress CD4+ T-cell proliferation and IFN-γ production in immunological non-responders. *In vitro* functional experiments indicated that inhibiting both PD-L1 and TGF-β pathways had a synergistic impact on restoring CD4+ T-cell activity. Therefore, Wang et al. called for a new therapeutic strategy by targeting PD-L1 and TGF-β pathways together to improve the immune recovery in immunological non-responders.

Xiao et al. from Beijing Youan Hospital, Capital Medical University presented a review on the characteristics, implications, and clinical significance of memory stem CD8^+^T cells in HIV/Mtb mono- and co-infection. Accumulating evidence has highlighted the significant role of CD8^+^T cells in both HIV and Mtb infections (Takata et al., 2022; Winchell et al., 2023), while research on memory stem CD8^+^T cells (CD8^+^T_SCM_) in HIV/Mtb co-infection is currently limited. The review discussed the function and potential mechanisms of interaction between HIV/Mtb co-infection and CD8^+^T_SCM_ cells, and prospected the development of immunotherapies and vaccines by targeting CD8^+^T_SCM_ cells.

Antiretroviral therapy (ART) has significantly reduced the mortality of people living with HIV, but the associated metabolic syndrome continues to be a significant challenge (Sears et al., 2019; Henning and Greene, 2023). Jin et al. developed and validated a nomogram to predict the risk of metabolic syndrome in people living with HIV receiving ART in China. The nomogram incorporated six parameters as predictive factors, which included age, ART regimen, body mass index, fasting blood glucose, high-density lipoprotein cholesterol, and HIV viral load. Calibration plots showed high consistency between the nomogram-predicted results and the clinically observed outcomes, while decision curve analysis confirmed the nomogram’s clinical applicability, which could effectively predict metabolic syndrome in people living with HIV following ART.

Although patients living with HIV and acute intracerebral hemorrhage (ICH) were observed in the early 1980s, data on ICH in HIV-infected patients remained limited. Huang et al. from Guangxi Medical University and Shantou University focused on this distinct population and investigated their clinical features and risk factors. They found that the majority of HIV-infected ICH patients were middle-aged (35 to 50 years of age) or elderly (60 years of age and older) men in the AIDS stage. Multivariate binary logistic regression analysis revealed that drug abuse, prolonged prothrombin time, and elevated triglyceride levels were independent risk factors for ICH and these combined predictors may serve as a valuable biomarker for predicting ICH. These findings would assist clinicians in implementing efficacious interventions by identifying a heightened susceptibility to ICH in people living with HIV.

Wang et al. from Capital Medical University investigated a risk model for the near-term prognosis of people living with HIV/AIDS and PCP and verified its effectiveness. Through this single-center, retrospective observational study, they established a PCP risk prediction model by incorporating five parameters, including ALB, PO2, TBIL, LDH, and CD4+ T lymphocyte count. During the evaluation, the model showed perfect discrimination with an AUC of 0.947. This convenient and easy-to-use model would assist clinicians in obtaining a determined probability value of PCP mortality with simple calculations to make more precise decisions regarding management strategies.

Wei et al. presented a rare case of an AIDS patient with thoracic SMARCA4-deficient undifferentiated tumors. The patient was a 69-year-old AIDS patient who initially presented with a fused, enlarged lymph node on the right clavicle and mild, unexplained pain under the right axilla that worsened with severe coughing episodes. By CT scan and laboratory tests, the patient was confirmed to have SMARCA4-deficient undifferentiated tumors. Considering the weak immune status, the patient was treated with first-line combination therapy of immunotherapy and anti-angiogenic drug instead of chemo-immunotherapy and achieved an overall survival for more than 22 months. This case report indicates that first-line treatment with a combination of immunotherapy and anti-angiogenic drugs may be a promising therapeutic strategy for patients with SMARCA4-deficient undifferentiated tumors.

Collectively, the research highlighted in this Research Topic underscores the importance of continuous input from the field that could contribute to the elimination of HIV/AIDS. We hope that this Research Topic will inspire scientists from different fields of research to focus on the pathogenesis and prevention of HIV/AIDS.
